# Effects of continuous renal replacement therapy on renal inflammatory cytokines during extracorporeal membrane oxygenation in a porcine model

**DOI:** 10.1186/1749-8090-8-113

**Published:** 2013-04-29

**Authors:** Hu Yimin, Yu Wenkui, Shi Jialiang, Chen Qiyi, Shen Juanhong, Lin Zhiliang, He Changsheng, Li Ning, Li Jieshou

**Affiliations:** 1 Department of General Surgery, Jinling hospital, medical school of Nanjing University, Nanjing, 210002, P.R. China; 2 Department of Anesthesiology, Jinling hospital, medical school of Nanjing University, No. 305, East Zhongshan Road, Nanjing, 210002, P.R. China

**Keywords:** Extracorporeal membrane oxygenation, Continuous renal replacement therapies, Renal inflammatory cytokines

## Abstract

**Background:**

Extracorporeal membrane oxygenation (ECMO) has been recommended for the treatment of patients with acute, potentially reversible, life-threatening respiratory failure which unresponsive to conventional therapy. But it is unclear about how ECMO affects renal tissue.

**Methods:**

Twenty-four piglets weighing 25 to 32 kg were used in this experiment. The piglets were randomly allocated to 4 groups of 6 animals each: sham group (S group), control group (C group), VV-ECMO group (E group), VV-ECMO combined with CRRT group (EC group). The piglets were sacrificed and the kidney tissue were harvest to determine the levels of IL-1β, IL-6, TNF-α and NF-КB by using the ELISA and RT-PCR method, respectively.

**Results:**

Compared with C group and S group, E group renal tissue IL-1β, IL-6, TNF-α and NF-КB expression increased significantly, respectively (*p* < 0.01). Compared with E group, EC group showed renal tissue IL-1β, IL-6, TNF-α and NF-КB expression decreased significantly, respectively (*p* < 0.05).

**Conclusion:**

ECMO enables to inflammatory cytokines including IL-1β, IL-6, TNFα, NF-КB released significantly, renal function impaired and immune homeostasis were to imbalance; ECMO combined with CRRT treatment can alleviate levels of inflammatory cytokines, maintain immune homeostasis balance and thus ameliorate the ECMO-related acute kidney injury(AKI).

## Background

Extracorporeal membrane oxygenation (ECMO) has been recommended for the treatment of patients with acute, potentially reversible, life-threatening respiratory failure which unresponsive to conventional therapy [1-3]. However, ECMO-related serious complications often lead to treatment failure because of long-term treatment support and more organs implicated. ECMO-induced acute renal dysfunction is a heavy burden for the society and a non-negligible problem [4,5]. Extracorporeal Life Support Organization (ELSO) Registry has indicated incidence of ARF during ECMO is up to 34.7% [6]. Nevertheless, the underlying mechanisms of acute renal failure during ECMO support are still unclear. Sell et al [7] have maintaned ECMO-induced acute renal dysfunction is mainly ascribed to pre-hypoxia and renal hypoperfusion. Gbadegesin et al [8] have suggested that hemolysis is a common complication during ECMO which may further impair renal function and delay renal recovery. However, kelly et al [9] have reported activation of cytolines might play a critical role in the changes of renal function. Continuous renal replacement therapies (CRRT) present significant advantages in terms of well clinical tolerance from a hemodynamic condition and blood purification. CRRT may provide effort to maintain optimal fluid status in patients receiving ECMO. It is an effective measure method in dealing with acute renal failure during ECMO support. Previous studies indicated that CRRT had been found to remove the inflammatory cytokines in both animal studies and clinical researches. This study was designed to investigate the expression of renal inflammatory cytokines by receiving concomitant ECMO and CRRT in the healthy swine model so as to better apply the extracorporeal life support technology for clinical patients.

## Methods

### Animals and groups

Twenty-four piglets weighing 25 to 32 kg were used in this experiment. All animals were used and cared followed “The Principles of Laboratory Animal Care” by the National Society of Medical Research and with the “Guide for the Care and Use of Laboratory Animals” (http://www.nap.edu/catalog/ 5140.html). This study was approved by the Animal Care Committee of Jingling Hospital.

The piglets were randomly allocated to 4 groups of 6 animals each: sham group (S group),control group (C group), VV-ECMO group (E group), VV-ECMO combined with CRRT group (EC group). The studies were done 24 hours.

### Surgical procedure

Anesthesia was induced with intramuscular injection of ketamine (20 mg/kg), diazepam (8 mg/kg), atropine (0.1 mg/kg) and maintained with intravenous ketamine (10-20 mg/kg/hr) and diazepam (8 mg/kg/hr). A 22-gauge intravenous (IV) catheter was then placed in the ear vein, and crystalloid fluid was administered at a rate of 7 to 10 mL/kg/h. After tracheotomy and an internal diameter 6.0 cm tracheal tube placement, mechanical ventilation was established, using volume-controlled mode with FiO_2_ of 21% and a positive end-expiratory pressure set at 5 mmHg throughout the experimental period. Tidal volumes were adjusted to 5-8 mL/kg. Respiratory rate was set at 15/min.

A venous catheter was placed into right internal jugular vein to administer Ringer’s lactate at a rate of 3 mL/kg/hr initially. The rate was increased to maintain the mean arterial pressure above 60 mmHg. The groin was instrumented via cutting down with a femoral arterial line for hemodynamic monitor and laboratory samples. A femoral venous line was placed for administration of intravenous fluids and medication. At the end of the experiment, the piglet was sacrificed with Potassium Chloride (40 mL, 1 mol/L,i.v.). The right kidney was removed rapidly and stored at -80°C for subsequent analysis.

### VV-ECMO proceduce

Before cannulation, each animal received bolus heparin (150 U/kg), followed by a continuous infusing of heparin to keep the activated clotting time (ACT) at 180 to 220 s during the process of ECMO. Right internal jugular vein was cannulated with a 17Fr Biomedicus arterial cannula (Medtronic Perfusion Systems, Minneapolis, MN), which was advanced into the right atrium. And another a 17 Fr Biomedicus venous drainage cannula (Medtronic Perfusion Systems, Minneapolis, MN) was inserted into right femoral vein. Placement of these cannulae was confirmed by ultrasound.

ECMO circuit (Quadrox PLS,Maquet,Germany) was primed with 500 ml Voluven and 200-300 ml Ringer’s lactate. The VV-ECMO system consisted of a centrifugal pump (Rotaflow Console, Maquet, Germany), and a heat exchange (Heater-Cooler Unit HCU 30, Maquet, Germany)maintaining temperature at 37°C. Sweep gas was 100% oxygen at a flow rate equal to the blood flow rate (1:1). Blood in circuit was drained from right femoral vein and infused into internal jugular vein at the rate of 50 ml/(kg.min).

### CRRT setting

CRRT was performed in a pre-dilution mode using a polysulfone membrane (1.4 m2 AV 600 s, ultraflux, Germany) connected to a continuous blood pump (Baxter BM 25, Baxter SAN Germany). Blood flow rate was set at 160-180 mL/min. A lactate-buffered replacement fluid (deploy Jingling prescription, china) was administered in a post-dilutional fashion.

Connecting CRRT device to ECMO circuit was performed in the following manner: The inlet (arterial) line of the CRRT circuit was connected after oxygenator by a three-way tap and the outlet (venous) line was connected to the circuit at another tap on infusing cannula. The treatment was zero balanced at an ultrafiltration rate of 20 mL/kg/h. The filters were unchanged during 24 hours each time.

### Determination of inflammation markers

Levels of TNF-α, IL-1β, IL-6 in renal tissues were quantified with enzyme-linked immunosorbent assay kits specific for the pig cytokines according to manufacturer instructions (R&D Systems, Munich, Germany). Values were expressed as picograms per milligram of protein for tissue samples. The nuclear extracts were prepared as described elsewhere. Electrophoretic mobility shift assay was performed with a commercial kit (Gel Shift Assay System; Promega Corporation, Madison, Wis). Nuclear factor КB (NF-КB) consensus oligonucleotide (AGT-TGA-GGG-GAC-TTT-CCC-AGG) was labeled with [γ-32P] adenosine triphosphate (Free Biotech, Beijing, China)with T4 polynucleotide kinase. Equal amounts of nuclear extract (60 μg) were added to 9 μL of gel shift binding buffer (10 mmol/L tris(hydroxymethyl) aminomethane hydrochloride, pH 7.5, 50-mmol/L sodium chloride, 0.5 mmol/L ethylenediaminetetraacetic acid, 1-mmol/L magnesium chloride, 0.5-mmol/L deoxythymidine triphos-phate, 4% glycerol, 0.05-mg/mL polynucleotide deoxyinosine deoxy-cytosine) for 15 min at room temperature. The mixture was incubated for 30 minutes with 1 μL of the phosphorus 32–labeled oligonucleotide probe. A 1 μL portion of loading buffer was added and the sample was electrophoresed in a 4% polyacrylamide gel at 390 V for 1 hour. The dried gel was exposed to x-ray film (Fuji Hyperfilm; Fuji Photo Film Co, Ltd, Tokyo, Japan) at 70°C. The intensity of the NF-КB complex was quantified by densitometry.

### Statistical analysis

Measurement data were expressed as mean ± SD. All the statistical analyses of the data were performed by a commercially available statistical software package (SPSS for Windows version 13.0; SPSS Inc, Chicago, IL).We used a repeated measurement analysis of variance (ANOVA with Repeated Measurements) between C group and S group, between S group, E group, EC group repeated measurement variables. *P* ≤0.05 was considered to be significant.

## Results

**Figure 1:**
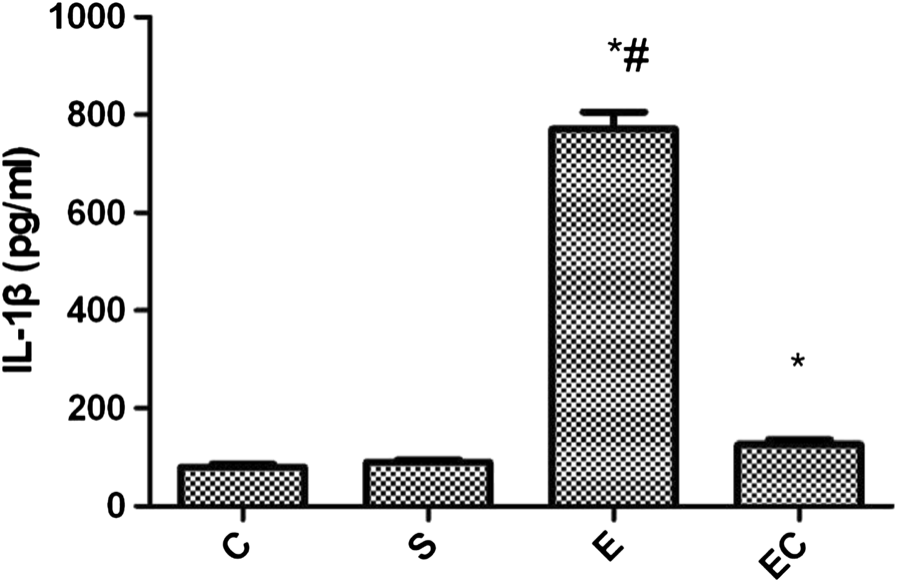
Renal IL-1β expression in all groups.

Compared with C group and S group, renal IL-1β expression in E group increased significantly(*p* < 0.01) (see Figure 1). Compared with E group, EC group showed decreased renal IL-1β expression(*p* < 0.01).

**Figure 2:**
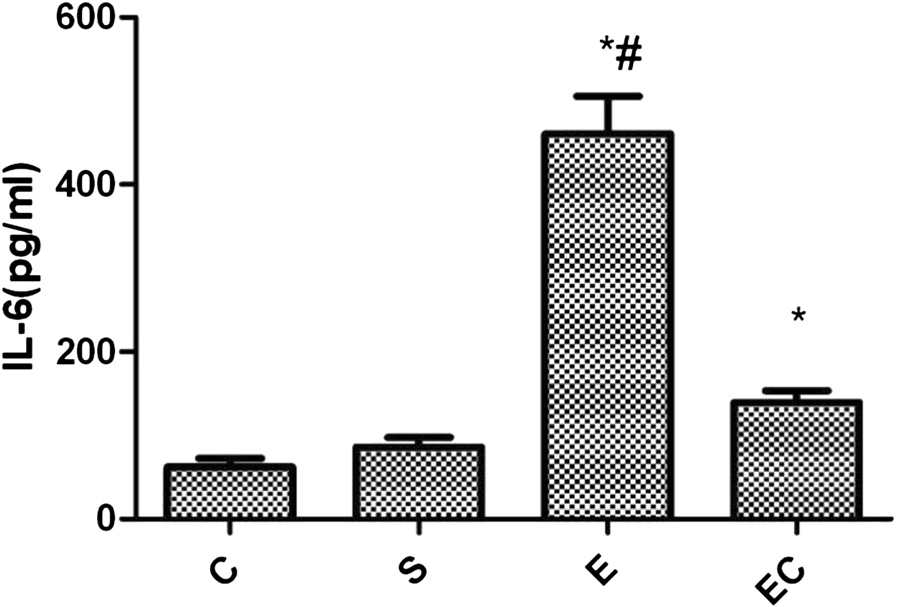
Renal IL-6 expression in all groups.

The expression of renal IL-6 in E group showed significant increase as compared with C group and S group(*p* < 0.01) (see Figure 2). Compared with E group, EC group showed renal tissue IL-6 expression decreased significantly(*p* < 0.01).

**Figure 3:**
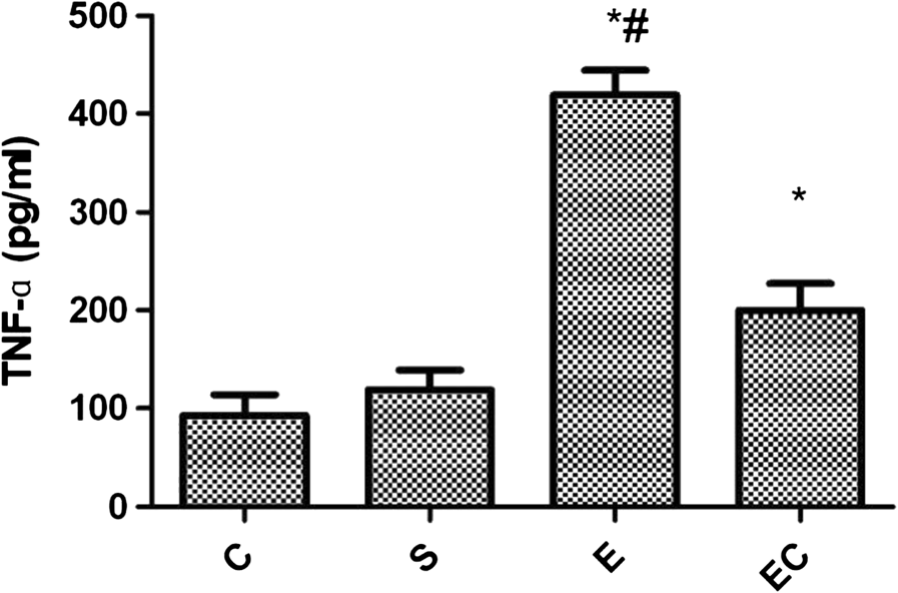
Renal TNF-α expression in all groups.

Compared with C group and S group, E group showed renal TNF-α expression significant increased(*p* < 0.01) (see Figure 3). Compared with E group, EC group showed renal TNF-α expression decreased significantly(*p* < 0.01).

**Figure 4:**
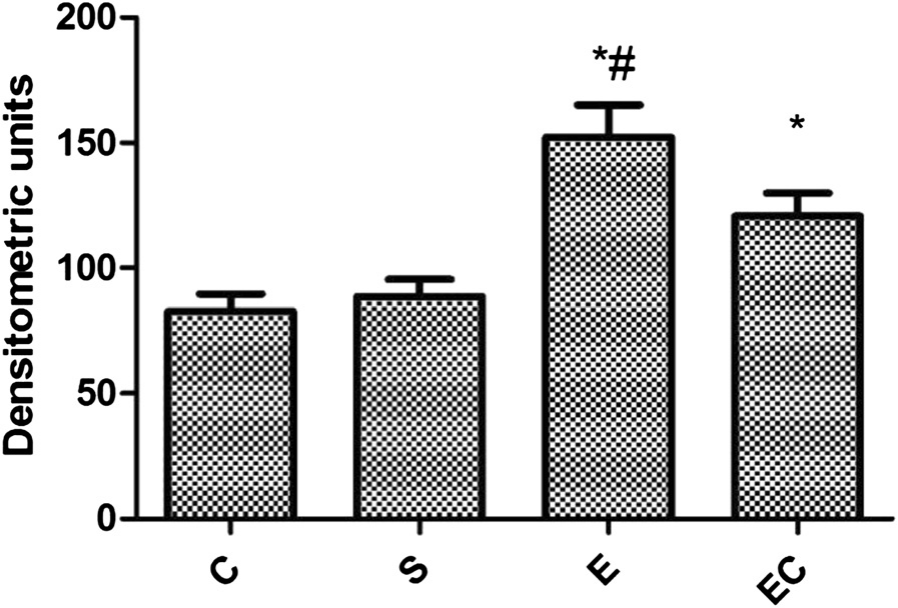
Renal NF-КB expression in all groups.

Compared with C group and S group, E group showed renal NF-КB expression increased significantly(*p* < 0.01) (see Figure 4). Compared with E group, EC group showed renal NF-КB expression decreased significantly(*p* < 0.05).

## Discussion

This study was designed to elucidate the expression of renal tissue cytokines by receiving concomitant ECMO and CRRT in the healthy swine model. Our results showed that it might remove ECMO-induced cytokines, maintain the role of immune equilibrium, reduce the extent of ECMO-related SIRS, thereby leading to improve ECMO-related acute renal dysfunction.

CRRT was applied to acute renal failure patients have been widely recognized. CRRT matched with human kidney by continuously, slowly and convection running. Its mainly advantage is that it could stabilize the hemodynamic, improve azotemia, electrolytes and water-salt metabolism, keep on cleaning cycle of all sorts of cytokines and improve nutrition [10-13].

Previous animal experiments and clinical studies have shown that blood components are continually exposed to non-biological artificial surface from cardiopulmonary bypass pipe and changes of the non-physiological hemorheology during ECMO running. This leads to the complement system, the kallikrein system, neutrophils and vascular endothelial cell activated, cell surface adhesion molecule expression, mast cell degranulation, tumor necrosis factor (TNFα), interleukin (*e.g.* IL-1β, IL-6, IL-8) and other cytokines signaling cascad style released,which triggered ECMO-related SIRS [14-17].

These cytokines amplified the response of neutrophils and endothelial cells, and which caused extensive microvascular injury, microcirculation acidic metabolites and a large number of oxygen free radicals which generated significantly. Anti-inflammatory and pro-inflammatory cytokines increased, inflammatory response was out of control and homeostasis of immune system was showed imbalance in plasma concentrations, These ultimately lead to multiple organ dysfunction, especially for the renal dysfunction. NF-КB is a key factor which leads to inflammation and acute kidney injury and it also can positively induce the synthesis of a variety of inflammatory cytokines.

The present study showed that inflammatory cytokines including IL-1β, IL-6, TNF-α, NF-КB significantly increased in the ECMO group, and further confirmed result that it can be evoked by ECMO-related SIRS when ECMO treatment used alone. Inflammatory cytokines including IL-1β, IL-6, TNFα, NF-КB also increased in the ECMO + CRRT group. However, compared with the ECMO group, both slowed down significantly. These indicated that the ECMO combined with CRRT treatment can exert an immunomodulatory effect in order to maintain the immune equilibrium. It also can further reduce the degree of ECMO-related SIRS, and thus play a significant role in reversing the occurrence of acute kidney injury which related with ECMO.

## Conclusions

The present data demonstrates that, by receiving concomitant extracorporeal membrane oxygenation and continuous renal replacement therapy could decrease inflammatory cytokine. ECMO enables to inflammatory cytokines including IL-1β, IL-6, TNFα, NF-КB released significantly, CRRT could also regulate the factors responsible for inflammation, including decreasing IL-1β, IL-6, TNF-α-mediated NF-κB pathway. This research provides a possible explanation to the molecular mechanism of improving renal function during combined ECMO and CRRT. Thus, the widely used combined ECMO and CRRT can alleviate levels of inflammatory cytokines, maintain immune homeostasis balance and thus ameliorate the ECMO-related acute kidney injury(AKI).

## Abbreviations

ECMO: Extracorporeal membrane oxygenation; CRRT: Continuous renal replacement therapies; ELSO: Extracorporeal life support organization; NF-КB: Nuclear factor КB; TNFα: Ttumor necrosis factor; IL-1β: Interleukin-1β; IL-6: Interleukin-6; IL-8: Interleukin-8; ACT: Activated clotting time; AKI: Acute kidney injury; SIRS: Systemic inflammatory response syndrome.

## Competing interests

The authors declare that there were no competing interests in this study. This study was supported by a grant for 12th five-year major project (No. AWS11j03).

## Authors’ contributions

H YM completed most the scientific word and drafted the manuscript. S JL, C QY performed the model of ECMO. S JL completed the statistic work. C QY, S JH, L ZL, H CS helped the first author to finish the study. Y WK, L N, L JS helped to conceptualize and design the study and L N was correspondence for the paper. All authors read and approved the final manuscript.
